# C-type lectin receptor agonists elicit functional IL21-expressing Tfh cells and induce primary B cell responses in neonates

**DOI:** 10.3389/fimmu.2023.1155200

**Published:** 2023-03-31

**Authors:** Maria Vono, Beatris Mastelic-Gavillet, Elodie Mohr, Malin Östensson, Josefine Persson, Thorunn A. Olafsdottir, Sylvain Lemeille, David Pejoski, Oliver Hartley, Dennis Christensen, Peter Andersen, Arnaud M. Didierlaurent, Ali M. Harandi, Paul-Henri Lambert, Claire-Anne Siegrist

**Affiliations:** ^1^ Center for Vaccine Immunology, Department of Pathology and Immunology, University of Geneva, Geneva, Switzerland; ^2^ Department of Microbiology and Immunology, University of Gothenburg, Gothenburg, Sweden; ^3^ Faculty of Medicine, Biomedical Center, University of Iceland, Reykjavik, Iceland; ^4^ Department of Pathology and Immunology, University of Geneva, Geneva, Switzerland; ^5^ Vaccine Adjuvant Research, Department of Infectious Disease Immunology, Statens Serum Institut, Copenhagen, Denmark; ^6^ Vaccine Evaluation Center, British Columbia (BC) Children’s Hospital Research Institute, University of British Columbia, Vancouver, BC, Canada

**Keywords:** T follicular helper (Tfh), interleukin-6 (IL-6), interleukin-21 (IL-21), vaccine adjuvants, C-type lectin receptor agonists, toll-like receptor agonists, neonatal vaccinology

## Abstract

**Introduction:**

C-type lectin receptor (CLR) agonists emerged as superior inducers of primary B cell responses in early life compared with Toll-like receptor (TLR) agonists, while both types of adjuvants are potent in adults.

**Methods:**

Here, we explored the mechanisms accounting for the differences in neonatal adjuvanticity between a CLR-based (CAF^®^01) and a TLR4-based (GLA-SE) adjuvant administered with influenza hemagglutinin (HA) in neonatal mice, by using transcriptomics and systems biology analyses.

**Results:**

On day 7 after immunization, HA/CAF01 increased IL6 and IL21 levels in the draining lymph nodes, while HA/GLA-SE increased IL10. CAF01 induced mixed Th1/Th17 neonatal responses while T cell responses induced by GLA-SE had a more pronounced Th2-profile. Only CAF01 induced T follicular helper (Tfh) cells expressing high levels of IL21 similar to levels induced in adult mice, which is essential for germinal center (GC) formation. Accordingly, only CAF01- induced neonatal Tfh cells activated adoptively transferred hen egg lysozyme (HEL)-specific B cells to form HEL^+^ GC B cells in neonatal mice upon vaccination with HEL-OVA.

**Discussion:**

Collectively, the data show that CLR-based adjuvants are promising neonatal and infant adjuvants due to their ability to harness Tfh responses in early life.

## Introduction

Early life immunization against infectious diseases remains a major public health need. The challenge comes from the difficulty to induce a potent immune response by a pre-mature immune system during early life, and the lack of knowledge on how to rationally design vaccines to induce protective immunity in the very young (1). Upon pathogen or antigen encounter, neonatal conventional dendritic cells (DCs) secrete low levels of IL12, leading to impaired type 1 T helper (Th1) responses (2) and higher levels of type 2 T helper (Th2) cell-associated cytokines, such as IL4 and IL13. Together with the anti-inflammatory cytokine IL10, this retains DCs in an immature state (3), impairing antigen presentation. In addition, delayed maturation of follicular dendritic cells (4) and inadequate development and expansion of T follicular helper (Tfh) cells (5, 6) result in the restricted induction of early life germinal center (GC) B cells, plasma cells and antibody responses (7).

Formulation of vaccines with adjuvants is a proven approach to improve their efficacy, but few adjuvants are yet used in infants. Aluminum salts, such as Aluminum Hydroxide (AH), are included in most infant vaccines and remain the only adjuvants approved for use in neonates (in hepatitis B vaccines). AH-containing vaccines only induce weak primary responses in early life but efficiently prime memory B cell responses, allowing stronger responses to booster doses (8). The same was observed in murine neonates, where AH-containing vaccines did not generate potent primary antibody responses (5). The candidate malaria vaccine RTS,S adjuvanted with the Toll-like receptor (TLR)-based adjuvant AS01(E) showed promising results in children above 6-weeks of age, but only when given in a 3-dose schedule spreading over several months (9). The oil-in-water emulsions MF59 and AS03 increased influenza vaccine responses in children above 6 months (10) but have not yet been tested in younger infants. In neonatal mice, an MF59-adjuvanted influenza vaccine failed to induce primary antibody responses (11).

TLR-ligands and C-type lectin receptor (CLR)-ligands have not yet been tested in human neonates. Both types of adjuvants showed an effect on activation of cord blood-derived human antigen-presenting cells (APCs) *in vitro* and increased immune responses in murine neonates *in vivo* (12, 13). Of note, responses to TLR-ligands increased with age (14–16). Given the need to develop more immunogenic vaccines for the neonates, it is critical to better understand and compare the modes of action of different adjuvants in early life.

GLA-SE is a squalene emulsion (SE) combined with the TLR4 agonist glucopyranosyl lipid adjuvant (GLA) (17). GLA-SE enhanced antibody and T cell responses when combined with an influenza antigen in preclinical adult models and in a human phase 1 study (18). In neonatal mice immunized against influenza, we found that GLA-SE enhanced Tfh cell responses but failed to generate primary GC structures and antibody responses (13). Similar results were generated following neonatal immunization with the TLR9 stimulating adjuvant IC31^®^ (13, 19).

Trehalose dibehenate (TDB) is a CLR agonist known to activate human newborn DCs *in vitro* (20) as well as neonatal murine DCs (21). CAF01 is composed of TDB incorporated in a liposomal delivery vehicle formed by the cationic surfactant dimethyldioctadecylammonium (DDA) (22). We recently demonstrated that CAF01, which is as potent as GLA-SE in adult mice (23, 24), successfully induced primary B cell responses in murine neonates (13). Curdlan, a different CLR agonist that binds receptor dectin-1, induced high and similar neonatal responses as CAF01 when formulated in DDA (13). Thus, it seems that the class of CLR-based adjuvants can circumvent a major limitation in early life immunization and could pave the way for the development of novel neonatal vaccines. Here, we addressed the mechanisms underlying the potent adjuvant effect of CLR-agonists on the neonatal immune system. We performed a detailed analysis of the Tfh, B cell and transcriptional signature of the CLR-activating adjuvant CAF01 in the LN draining the vaccination site compared to the TLR4-based adjuvant GLA-SE in neonatal mice. GLA-SE was selected for the comparative analysis because it has neonatal adjuvanticity (13) and previous studies comparing the adjuvanticity of CAF01 and GLA-SE in adult mice are available (23, 24).

## Materials and methods

### Mice

Adult CB6F1/OlaHsd, BALB/c OlaHsd and C57BL/6J OlaHsd were purchased from Harlan (Horst, The Netherlands). Female BALB/c OlaHsd and male C57BL/6J OlaHsd mice were crossed to produce F1 CB6F1 mice. SW_HEL_ mice (C57BL/6 CD45.1) (25) were obtained from Robert Brink, and neonatal C57BL/6J mice were also generated in house. Both female and male neonatal mice were used in the experiments. All mice were bred, kept in specific pathogen-free facilities in accordance with local guidelines and used at 1 week (neonates) or 6-8 weeks (adults) of age. Typical litter size varies from 6 to 8 neonatal mice, consequently the number of neonatal mice allocated to each group may slightly differ between experiments. All animal experiments were approved by the Geneva veterinary office (authorization GE/05/16) and conducted under relevant Swiss and European guidelines.

### Antigens, adjuvants, and immunization

A monovalent purified subunit influenza HA vaccine from H1N1 A/California/7/2009 (provided by the former Novartis Vaccines Unit) was used for all influenza model experiments (hereafter referred to as HA). Groups of 5 to 8 CB6F1 neonatal and adult mice were immunized subcutaneously (s. c.) with 100 µl of HA (1 μg) alone or in combination with either CAF01 (250 μg DDA/50 μg TDB, Statens Serum Institut, Copenhagen, Denmark), or GLA-SE (5 μg GLA and 2% v/v squalene, Infectious Diseases Research Institute, Seattle, WA, USA).

Mice were immunized at the base of the tail and inguinal draining lymph nodes (LNs) were harvested.

### RNA extraction for microarray experiments

Neonatal mice were sacrificed at 1 and 7 days following a single vaccination with either HA/PBS, HA/CAF01 or HA/GLA-SE. Inguinal draining LNs were collected in 1.5 mL RNA later buffer (Qiagen) in low binding micro tubes (Sarstedt) and left at 4°C over night before transfer to -80°C.

RNA was extracted from dLNs using the RNeasy Mini QIAcube kit (Qiagen), according to the manufacturer’s instructions and as already described (Olafsdottir T.A. et al., 2016). Spectrophotometry was used to measure RNA concentration (ND-1000 spectrophotometer, NanoDrop Technologies Inc., USA) and the RNA integrity number (RIN), an indicator of sample quality, was determined using an Agilent 2200 TapeStation and 2100 Expert Software (Agilent Technologies, USA). Only samples with good quality RNA, defined as having a 260/280 ratio approximating 2 and RIN >7, were used in microarray analyses.

### Microarray analysis and data acquisition

Whole genome microarray analysis was performed at the bioinformatics and expression analysis core facility, Karolinska Institute, Sweden using an Illumina platform according to the manufacturer’s protocols. Briefly, the RNA was labeled with a fluorescent linear amplification kit according to manufacturer’s instructions. The quantity and labeling efficiency were verified before the samples were hybridized to whole-genome 8 × 60 k mouse expression arrays, which were scanned at 5 μm using an Agilent scanner. Image analysis was performed with Feature Extraction software (version 11.5.1.1, Agilent Technologies) to generate raw microarray data.

The raw data was pre-processed and normalized using the limma package (26) and corrected for background using the “normexp” method in the statistical program R (R Core Team (2020). R: A language and environment for statistical computing. R Foundation for Statistical Computing, Vienna, Austria. URL https://www.R-project.org/). Differentially expressed genes (DEGs) compared to baseline were identified by performing the moderated Student’s t-test at each of the time points (*p*-value) followed by further adjustments for multiple testing using the Benjamini–Hochberg method (adjusted *p*-value). Changes in expression induced by the adjuvants were calculated by dividing the log2 expression value of each individual adjuvant-treated group by the log2 expression value of the group receiving HA alone at any given time point. The microarray data have been deposited to the Gene Expression Omnibus under accession number GSE226513.

**Gene set enrichment analysis (GSEA):** All annotated pathways for *Homo sapiens*, *Mus musculus*, *Rattus norvegicus*, *Danio rerio*, *Sus scrofa* and *Saccharomyces cerevisiae* available on WikiPathways database (27) were used to generate gene sets, as well as the KEGG pathway database (28) relative to GRCm38.89. Genes were ranked by their calculated fold-changes (decreasing ranking). A gene set analysis using the GSEA package version 2.2 (29, 30) from the Broad Institute (MIT, Cambridge, MA) was used to analyze the pattern of differential gene expression between the two groups. Gene set permutations were performed 1000 times for each analysis. The Normalized Enrichment Score (NES) was calculated for each gene set. GSEA results with a nominal FDR < 0.05 and abs (NES) > 1 were considered significant.

**Heatmaps of leading-edge genes:** Genes appearing at least twice in all core-enrichment of pathways with abs (NES) > 1 were selected and organized in categories. Fold-changes in each comparison were used for hierarchical clustering using the function hclust in R.

### Semi-quantitative real-time PCR

RNA and cDNA were prepared from the 2 inguinal draining lymph nodes or from the indicated FACS-sorted cell populations as previously described (5). Briefly, RT-PCRs were performed on a SDS 7900 HT instrument (Applied Biosystems). In each reaction, *GusB*, *EEf1*, and *MmRPS9* were used as internal control genes for data normalization (5). Raw cycle threshold values obtained with SDS 2.2 (Applied Biosystems) were imported in Microsoft Excel, and normalization factor and fold changes were calculated using the GeNorm method (31). The primers used in this study for real-time PCR are shown in **Supplementary Table 1**.

### *In vitro* HA restimulation of dLNs cells and cytokine assessment by bioplex analysis

The two inguinal draining LNs of each mouse were pooled and processed to obtain single-cell suspensions, and 1x10^6^ cells were restimulated with HA (1μg/ml) or incubated in medium alone for 72h at 37°C, 5% CO_2_. Supernatants were analyzed for IL-6, IL-4, IL-10, IL-2, IFN-γ, IL-17, TNF-α, IL-1b, CXCL10, CXCL11, KC, MCP-1, MIP1a, MIP1b, CXCL2, CXCL5, and IL-21 by multiplex-bead ELISA assays, according to the manufacturer’s instructions. To assess all these analytes, we used a Bio-Plex Pro™ Mouse Chemokine Panel 33-Plex, a custom Bio-Plex (Biorad) and a custom ProcartaPlex (Invitrogen). The plates were read on the Bio-Plex^®^ MAGPIX™ Multiplex Reader (Biorad) and cytokine concentrations were determined by 4-parameter logistic non-linear regression analysis of standard curve.

### Flow cytometric analysis of lymph node cells for phenotyping of Tfh cells and GC B cells

Cells from the two draining LNs of each individual mouse were pooled and stained with combinations of the following antibodies to phenotype Tfh cells: α-CD4-PacificBlue, α-ICOS-PECy7, α-CD8-APCCy7 (all from Biolegend), α-B220-PE-CF594, and α-Bcl6-Alexa647 (BD Biosciences), and α-PD1-PE (eBioscience). CXCR5 staining was performed using purified rat anti-mouse CXCR5 (BD Biosciences), followed by FITC anti-rat IgG (Southern Biotech), and normal rat serum (eBioscience). Intracellular Bcl6 staining was performed with the Foxp3 Staining Set (eBioscience). To phenotype GC B cells, cells were stained with fluorescently labeled antibodies to GL7, B220, CD95, TCR-β, CD8, (Fas) (all from BD Biosciences), PD-1, Ter119, GR1, CD11c (all from eBioscience), and CD4 (from BioLegend). The stained cells were analyzed using a Gallios cytometer (Beckman Coulter) and the generated data analyzed using FlowJo Software (Tree Star). 

### Enzyme-linked immunosorbent assay of HA-specific IgG antibodies

Mice were bled from the tail vein at the indicated time points except for neonatal mice at day 0 that were bled by decapitation. Titration of HA-specific total IgG antibody titers was performed by ELISA on individual serum samples as previously described (11, 13).

### Isolation of distinct T cell subsets by flow cytometry sorting

One week-old (8 mice/group) and adult CB6F1 mice (6 mice/group) were immunized s.c. at the base of the tail with either HA/CAF01 or HA/GLA-SE. Ten days post vaccination the 2 inguinal draining LNs were collected, samples from each group were pooled and CD4^+^ T cells were enriched by using the EasySep™ Mouse CD4^+^ T Cell Isolation Kit (Stemcell Technologies). Total CD4^+^ T cells were stained with fluorescently labeled antibodies to CD4, B220, PD-1, CXCR5, and streptavidin. CXCR5 staining was performed using purified anti-CXCR5 (BD Pharmingen), followed by FITC anti-rat IgG (Southern Biotech), and normal rat serum (Invitrogen). DAPI was added before sorting as a dead cell discriminator dye.

Highly pure CD4^+^CXCR5^high^PD-1^high^ Tfh cells, CD4^+^CXCR5^dim^PD-1^dim^ effector T cells and the CD4^+^CXCR5^neg^PD-1^neg^ resting T cells (non- Tfh) were simultaneously isolated from the enriched CD4^+^ T cells by flow-cytometry sorting using a MoFlo^®^ Astrios™ flow cytometer (Beckman Coulter); (purity ≥99%).

### Adoptive transfer experiment

For transfer experiments, we used SWHEL mice whose B cells carry prearranged heavy and light chains forming immunoglobulins that recognize an epitope of HEL (25). Adult SWHEL CD45.1+ B cells were purified from spleens and LNs by negative selection using magnetic beads (Miltenyi Biotec). B cell purity was ≥ 90%. The percentage of HEL+ B cells, about 13% of total transferred B cells, was detected using the HyHEL9 monoclonal antibody conjugated to AlexaFluor^®^ 647 (25). We transferred 2x10^6^ SWHEL CD45.1+ adult B cells per neonatal mouse intraperitoneal (i.p.). Twenty-four hours post transfer recipients and control neonatal mice were immunized s.c. at the base of the tail with 100 µl of HEL-OVA (20 μg) alone or in combination with either CAF01 (250 μg DDA/50 μg TDB) or GLA-SE (5 μg GLA and 2% v/v squalene). The strategy of HEL-OVA conjugation was adapted from Qi et al. (32). HEL-OVA was produced using a bis-arylhydrazone conjugation approach (33). Briefly, OVA was derivatized with a 5-fold molar excess of 6-hydrazinonicotinic acid acetone hydrazone, and HEL was derivatized with succinimidyl 4-formylbenzoate (at equimolar ratio). The two derivatized proteins were reacted together (HEL at a 10-fold molar excess with respect to OVA) for three hours under non-denaturing conditions, and the resulting protein-protein conjugate was isolated by size exclusion chromatography.

### Flow cytometric analysis of lymph node cells from the adoptive transfer experiments

Cells from the two draining LNs of each individual mouse were pooled and stained with combinations of the following antibodies: α-CD45.1-BV510, α-CD138-BV421 (all from Biolegend), α-B220-PE-CF594, α-GL7-FITC, α-CD95(Fas)-PECy7 (all from BD Biosciences), α-PD1-PE, and α-CD4-PerCpCy5.5 (all from eBioscience).

CXCR5 staining was performed using purified rat anti-mouse CXCR5 (BD Biosciences), followed by FITC anti-rat IgG (Southern Biotech), and normal rat serum (eBioscience).

HEL-specific B cells were detected by intracellular staining using BD Cytofix/Cytoperm kit (BD Biosciences). Cells were first incubated with 200 ng/ml of HEL followed by Alexa647-conjugated anti-HEL antibodies (clone HyHEL9). The stained cells were analyzed using a Fortessa cytometer (BD Biosciences) and the data were analyzed using FlowJo Software (Tree Star).

### Enzyme-linked immunosorbent assay to detect HEL-specific IgG antibodies

Mice were bled at the time of sacrifice, day 12 post-injection, and blood was kept at 4°C for 1 hour prior to serum collection. For HEL-specific ELISA, MaxiSorp plates (Nunc) were coated with 10 μg/ml of HEL (Sigma-Aldrich) in PBS overnight at 4°C. Plates were washed three times with 0.05% Tween in PBS and blocked with 1% BSA in PBS for 1 hour at 37°C. After washings, plates were incubated for 1 hour at 37°C with individual mouse sera. Plates were washed and incubated for 1 hour at 37°C with an HRP goat anti-mouse IgG (Novex). After washings, ABTS Enhancer (Sigma-Aldrich) was added for peroxidase detection. Color reaction was measured with SoftMax (Molecular Devices) reader by determining OD at 405 nm. IgG1 titers were measured similarly, except that an HRP rat anti-mouse IgG1 antibody (BD Pharmingen) was used. To detect IgG2c titers an HRP rabbit anti-mouse IgG2c antibody (Southern Biotech).

### Statistical analysis

Data were analyzed using Prism 9.0 (GraphPad Software) and presented as mean ± standard error of the mean (SEM) of at least 3 independent experiments. Difference between groups was analyzed as described in figure legends. *P* values less than 0.05 were considered statistically significant.

## Results

### Transcriptomic profiling of dLNs from neonatal mice immunized with HA/CAF01 or HA/GLA-SE

**Figure 1:**
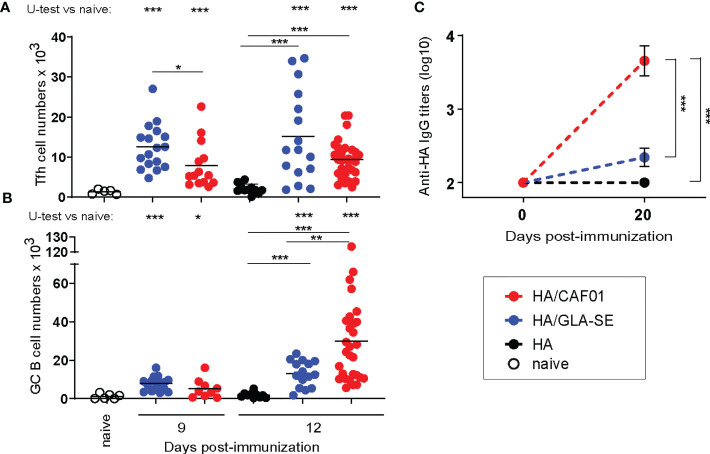
Neonatal primary Tfh, germinal center B cell, and antibody responses to HA/CAF01 or HA/GLA-SE. Groups of neonatal CB6F1 mice were immunized s.c. with HA formulated with CAF01 or GLA-SE. Age-matched mice receiving HA/PBS or non-injected (naïve) were used as control. **(A, B)** Draining LNs were harvested at the indicated time points after immunization, and samples were analyzed by flow cytometry. Graphs report the numbers of **(A)** T follicular helper (Tfh) cells and **(B)** Germinal Center **(GC)** B cells. Dots show values per individual mouse, whereas black lines indicate means. **(C)** HA-specific IgG antibody titers in sera collected before (day 0) and 3 weeks post-immunization (day 20). Values represent mean logarithmic titers (log 10) of five to eight mice per group ± SEM. **(A-C)** Data were pooled from at least two independent experiments per time point. Statistical analysis were performed using the Mann-Whitney U test: *P < 0.05, **P < 0.01, ***P < 0.001.

We previously reported that CAF01 adjuvant elicited significantly higher and sustained primary neonatal antibody responses to HA compared to GLA-SE (13). HA/CAF01 and HA/GLA-SE both significantly increased primary neonatal Tfh cells when compared to immunization with HA alone. However, only HA/CAF01 induced neonatal B cell differentiation into GC structures (13) and antibody production after a single dose (**Figures 1A-C**). Higher Tfh cell numbers were occasionally observed in neonatal mice receiving HA/GLASE (**Figure 1A**) but this was not consistent over experiments, and it is likely due to the high inter-animal variability.

To explore the mechanisms accounting for the differences in potency between CAF01 and GLA-SE adjuvants, we performed genome-wide transcriptomic analysis. RNA was extracted from LNs of neonatal mice draining the injection site at 24 hours and 7 days after injection of HA alone (control), or adjuvanted with either CAF01 or GLA-SE. These two time points were selected to include early innate (24h) and adaptive (7 days) immune responses. Gene expression was analyzed using genome-wide microarray analysis. The data were subjected to a ranked gene set enrichment analysis (GSEA), followed by leading edge analysis, revealing the subset of genes (the leading-edge subset) which contributed the most to the enrichment signal (30).

In a first round of analysis, we focused on the changes in gene expression between each adjuvant group and the HA alone group (logFC were calculated using HA group as the reference).

**Figure 2:**
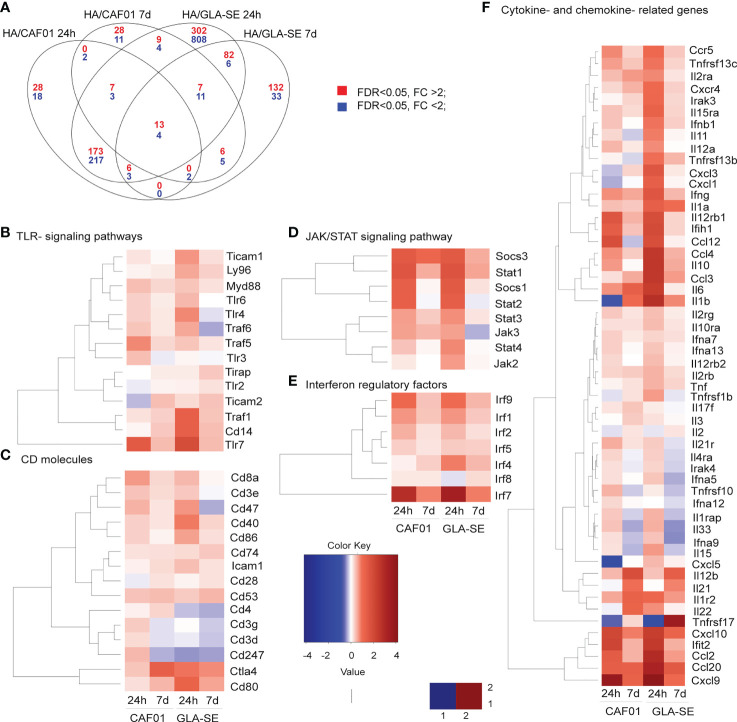
Transcriptomic profiling of whole draining LNs from neonatal mice responding to HA/CAF01 or HA/GLA-SE. Groups of neonatal CB6F1 mice were immunized s.c. with HA alone or formulated in combination with CAF01 or GLA-SE. Two draining inguinal LNs were collected 24 h and 7 days post-immunization to perform microarray analysis. **(A)** The Venn diagram depicts the numbers of unique and shared differentially expressed genes (DEGs) between either adjuvant group compared with the HA alone group, at 24 h and 7 days post immunization as indicated. Blue and red numbers indicate the DEGs that were significantly downregulated (blue) and upregulated (red), respectively. **(B–F)** Heat maps show the expression profiles of selected genes associated with **(B)** TLR signaling pathways, **(C)** genes encoding CD molecules, **(D)** JAK-STAT signaling pathways, **(E)** genes encoding interferon regulatory factors, and **(F)** cytokine- and chemokine-related genes, after CAF01 or GLA-SE at the indicated time points.

The Venn diagram indicates the overlap of genes that were significantly up-regulated (red) or down-regulated (blue), after HA/CAF01 or HA/GLA-SE immunization (**Figure 2A**). Genes appearing at least twice in all core-enrichment of pathways with abs_(NES) > 1 were selected and organized in heatmaps. Overall, HA/GLA-SE induced more changes in gene expression than HA/CAF01 when compared to HA alone (**Figure 2A** **)**. However, we observed a high degree of similarity in transcriptional changes of genes related to immune responses induced by HA/CAF01 or HA/GLA-SE, compared with HA alone. The changes in gene expression were more evident 24 h post-injection, but many persisted until day 7 post-injection (**Figure 2**, and **Supplementary Figures 1**, **2A**). Heat maps show the expression profiles of all identified differentially expressed genes (DEGs) grouped into functional categories (**Figures 2B-F**, and **Supplementary Figure 1**). Significant changes were observed with both adjuvants in cytokine and chemokine-related genes, genes involved in JAK-STAT signaling pathways, genes coding for CD molecules or interferon regulatory factors (**Figures 2B-F**), genes coding for oligoadenylate synthase (OAS) proteins, mitogen-activated protein (MAP) kinases, complement components, apoptosis-related genes, and MHC-associated genes (**Supplementary Figure 1**, **2A**).

However, some pathways were specific to each adjuvant. At 24 hours post-injection, GLA-SE specifically up-regulated genes belonging to the TLR4 signaling pathway, e.g. *TLR4*, *CD14*, and *LY96*, revealing that the GLA-induced TLR4 signaling pathway can be activated even in neonatal mice. At day 7 post-injection, only GLA-SE up-regulated the transcription of *IL10* gene while CAF01 specifically up-regulated the expression of *IL6* gene.

**Figure 3:**
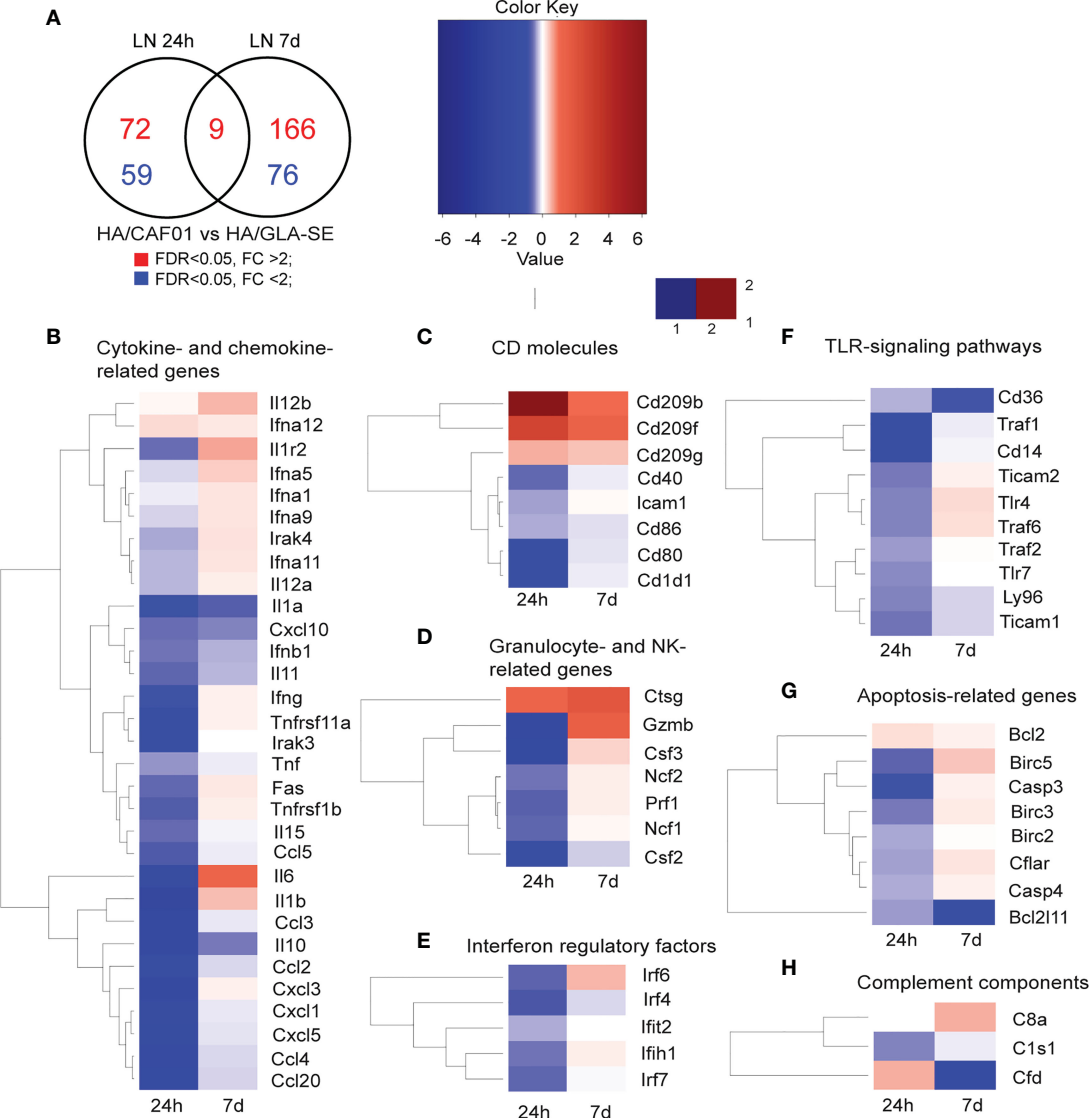
Co-expression analysis in dLNs revealed shared and unique features of CAF01 and GLA-SE adjuvan**ts.** Groups of neonatal CB6F1 mice were immunized s.c. with HA alone or formulated with CAF01 or GLA-SE. Transcriptomic profiles resulting from the comparison HA/CAF01 versus HA/GLA-SE are shown. **(A)** Venn diagram depicts the numbers of genes that were significantly up-regulated (red) or down-regulated (blue) in the HA/CAF01 group compared with the HA/GLA-SE group in inguinal draining LNs at 24 h and 7 days post immunization. **(B–H)** Heat maps show the expression profiles of selected genes associated with **(B)** cytokine and chemokine activity, **(C)** encoding for CD molecules, **(D)** granulocyte-associated genes, **(E)** interferon regulatory factors, **(F)** TLR signaling genes, **(G)** apoptosis-related genes and **(H)** complement components.

Differences were further highlighted by performing a direct comparison between CAF01 and GLA-SE adjuvants. The Venn diagram shows the genes that were significantly up- or down-regulated in the comparison of HA/CAF01 versus HA/GLA-SE (**Figure 3A**). Heat maps show genes appearing at least twice in all core-enrichment of pathways with abs_(NES) > 1 following leading-edge analysis. The majority of the significantly altered genes were organized in biological functional categories (**Figures 3B-H**), and the remaining ones are shown in **Supplementary Figure 2B**.

GLA-SE induced stronger early responses in the draining LN than CAF01. At 24 h post-injection, GLA-SE induced higher expression levels of genes coding for cytokines, chemokines, genes belonging to TLR- signaling pathways, interferon regulatory factors, co-stimulatory molecules such as *CD40*, *CD80*, and *CD86*, and genes like *ICAM1*, perforin (*PRF1*), granzyme B (*GZMB*), and the colony stimulating factors 2 and 3 (*CSF2* and *CSF3*). At day 7 post-injection, only a few genes were more expressed in the GLA-SE group, including *IL10*, *IL1α, IFNB1*, *IL11, TNF*, and several chemokines, including *CXCL10* (**Figure 3B**). Further, the co-stimulatory molecules *CD40*, *CD86*, and *CD80* were more expressed after GLA-SE use at day 7 post-injection compared to CAF01 use (**Figure 3C**).

At 24h post-injection, CAF01 induced higher expression of a few genes. Among these were DC-SIGN (*CD209B*, *CD209F*, *CD209G*), cathepsin G (*CTSG*), and several enzymes (**Figure 3** **and** **Supplementary Figure 2B**). Importantly, at day 7 post-injection, CAF01 exclusively up-regulated *IL6* (**Figures 2F**, **3B**), as reflected by its top position in the list of leading-edge genes. *IL12B*, *IL1β, IL1R2*, *IFNG* were expressed at higher levels by CAF01 as compared to GLA-SE (**Figure 3B**). *DC-SIGN*, *CTSG* and *GZMB* were highly expressed in the CAF01 group also at day 7.

Interestingly, *PPARg* was expressed at higher levels following HA/GLA-SE injection at both time points (**Supplementary Figure 2B** **)**. *PPARg* is a member of the peroxisome proliferator-activated receptor family. In adults, PPARg promotes regulatory T cell survival and inhibits the formation of Tfh cells and GC reactions *via* the regulation of Bcl6 and IL21 (34).

In summary, the transcriptomic analysis revealed that at 24 h post-injection both CAF01 and GLA-SE induced changes in genes associated with innate responses with GLA-SE eliciting stronger early responses than CAF01. At day 7 post-injection, CAF01 exclusively up-regulated *IL6*, induced higher levels of *IL12B*, and lower levels of *PPARg* than GLA-SE. In contrast, GLA-SE increased *IL10* levels. In adults, all the genes encoding these cytokines have a well-established role in T cell differentiation, with IL6 playing a key role in Tfh differentiation and GC B cell responses. Therefore, the higher level of IL-6 is in keeping with the enhanced formation of GC and antibody responses observed with HA/CAF01 in neonates. IL10 suppresses the production of pro-inflammatory cytokines by DCs and macrophages. Inhibition of IL12 by IL10 prevents APCs from inducing Th1 cell differentiation. These findings suggest that the different microenvironment in the dLN could lead to differences in the quality of the T cell responses elicited by CAF01 and GLA-SE and explain the ability of CAF01 to elicit primary B cell responses in neonates.

### Differential cytokine milieu is induced by CAF01 and GLA-SE in the dLNs of neonatal mice concomitant the type of Th responses

Next, we sought to ascertain the microarray results on the levels of the main genes differentially expressed between CAF01 and GLA-SE by quantitative real-time PCR at day 7 and day 10 post-injection. The genes whose expression levels were assessed include *IL6, IL12B, IL10, IL1β, IL4, IL13, IL21, GZMB, TBX21, IFNγ, IL17, IL2, DC-SIGN, CTSG.*

**Figure 4:**
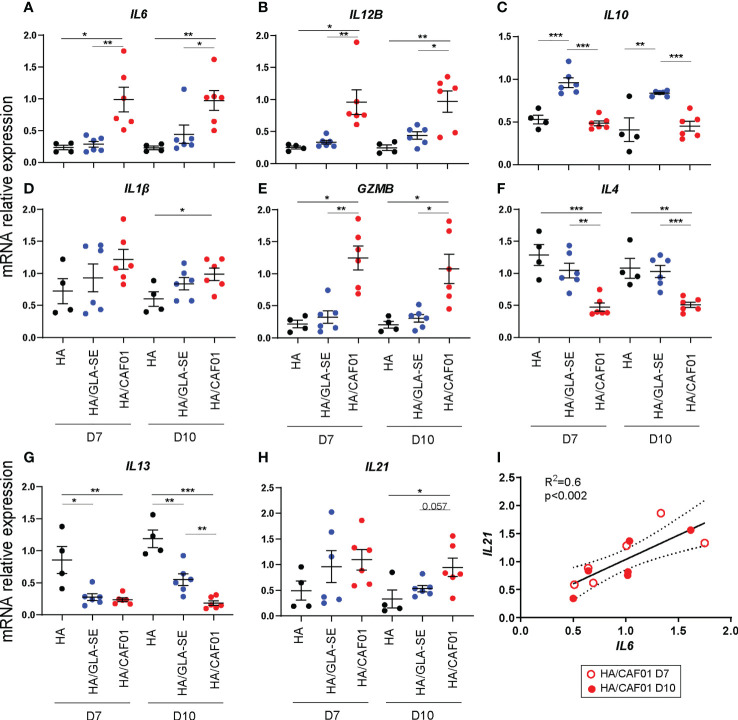
CAF01-elicited neonatal adaptive responses develop in an IL6-rich environment. **(A-H)**. One week-old CB6F1 mice were immunized s.c. with HA/PBS, HA/CAF01 or HA/GLA-SE and the two draining LNs were collected at day 7 or 10 post-immunization. RNA from total dLNs was used to measure the expression levels of selected genes by RT-PCR. mRNA expression levels of **(A)** *IL6*, **(B)** *IL12B*, **(C)** *IL10*, **(D)** *IL1B*, **(E)** *GZMB*, **(F)** *IL4* and **(G)** *IL13*, and **(H)** *IL21* are shown. Dots show values per individual mouse (N ≥ 4 per group) whereas black bars indicate means ± SEM. Statistical analysis were performed using the Mann-Whitney U test: *P < 0.05, **P < 0.01, ***P < 0.001. **(I)** Graph showing the correlation between the mRNA expression levels of *IL6* and *IL21* in dLN of neonates receiving HA/CAF01; data from day 7 (open circle) and 10 (filled circle) post-immunization were pooled. N = 6 mice/group; Data were pooled from two independent experiments per time point.

CAF01 significantly increased *IL6* and *IL12B* expression in dLNs at day 7. GLA-SE exclusively increased *IL10* gene expression. Importantly, comparable results were observed at day 10 post-injection. *IL1β* levels did not differ between HA/CAF01 and HA/GLA-SE **(** **Figures 4A-D**). Further, higher levels of transcripts for *GZMB* and *CTSG* were observed in the dLNs of the CAF01 group, while the transcript level of *DC-SIGN* did not show a significant change (**Figure 4E**; **Supplementary Figure 3**).

In adults, IL6 and IL12 are known to favor the induction of Th1 cell responses that are difficult to elicit in neonates due to a preferential polarization of the T cell response toward Th2, characterized by high levels of IL4 and IL13. CAF01 induced lower *IL4* levels compared to both HA alone and HA/GLA-SE. Both adjuvants induced lower *IL13* levels than HA alone at day 7, but at day 10 HA/GLA-SE induced significantly higher levels of *IL13* than HA/CAF01 (**Figures 4F, G**). In agreement with these data, we observed higher expression of the transcription factor *TBX21*, which regulates Th1 responses, following HA/CAF01 immunization. Nevertheless, similarly low levels of IFNγ gene expression were detected in dLNs of neonatal mice with both adjuvants (**Supplementary Figure 3**).

IL6 was originally isolated as a B-cell differentiation factor. It is also required for IL21 production by CD4+ T cells, and Tfh cell generation, and it provides survival signals for post-mitotic plasma cells (35–39). IL21 is a key cytokine for Tfh functionality and is required for GC-Tfh development and the subsequent B cell responses, which develop in neonatal mice only following HA/CAF01 immunization (13). IL21 is mainly produced by Tfh cells, of which the responses peak around day 10 post-injection with either of the two adjuvants in neonates (13). Of note, at day 10 post vaccination, only HA/CAF01 significantly increased the levels of *IL21* in the total dLNs when compared to HA alone **(** **Figure 4H** **)**. Importantly, in mice receiving HA/CAF01, *IL6* level correlated with *IL21* level at both time points **(** **Figure 4I** **)**.

**Figure 5:**
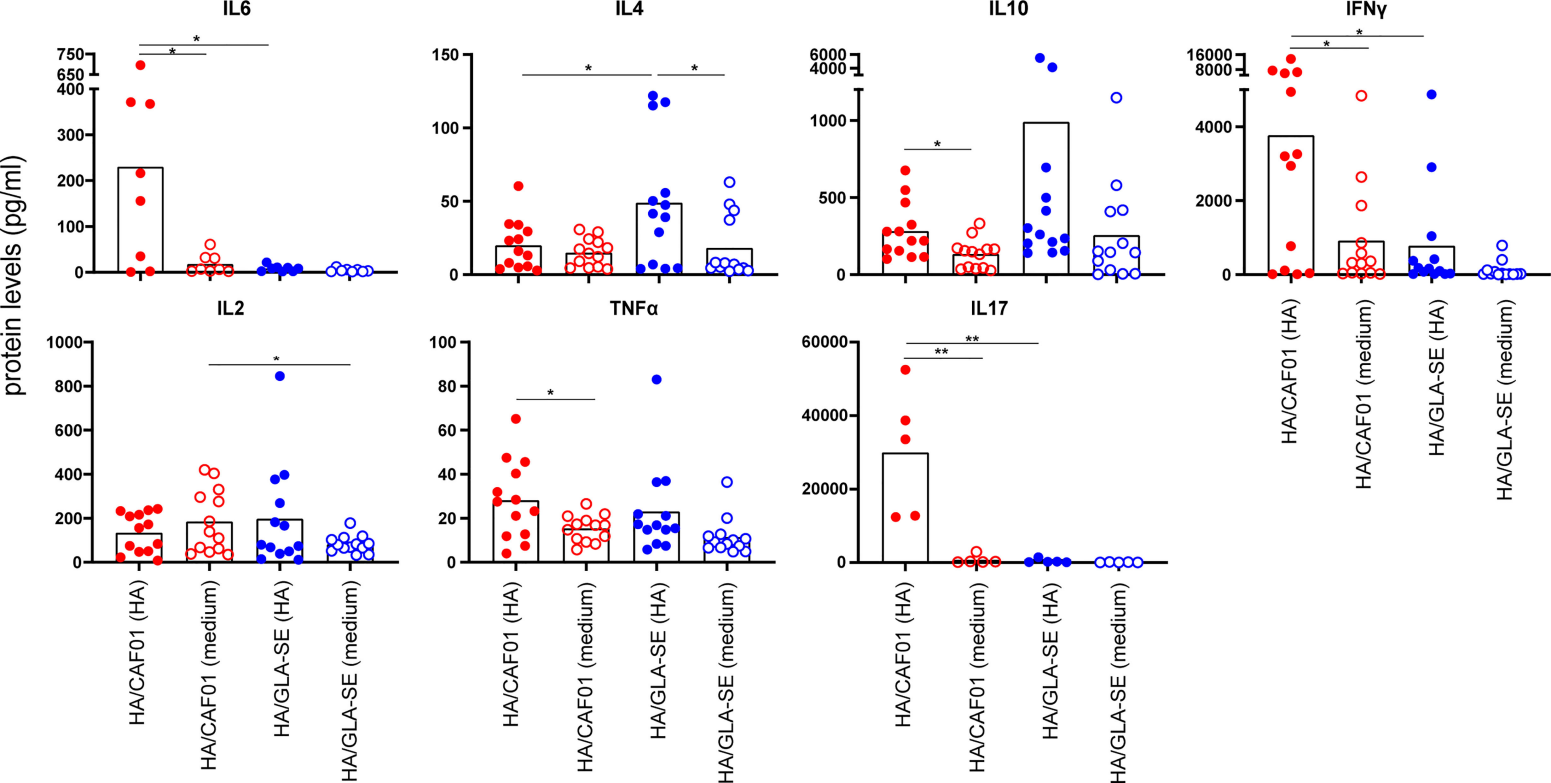
At day 10 post immunization CAF01 induced high levels of IL6 in draining LNs and higher levels of IFNγ and IL17 than GLA-SE upon HA restimulation ex vivo. Neonatal CB6F1 mice received HA/CAF01 or HA/GLA-SE and dLNs were collected 10 days after immunization and mashed to prepare single cell suspensions. Cytokine secretion in the supernatants was detected by luminex immunoassay after 3 days of restimulation with HA antigen or medium only. Values are expressed as picograms per milliliter (pg/ml); dots show values per individual mouse (N ≥ 5 per group) whereas black bars indicate means. Statistical analyses were performed using the Mann-Whitney U test: *P < 0.05, **P < 0.01. Data were pooled from at least two independent experiments.

In agreement with our results at the mRNA level, high levels of IL6 protein were observed only from cells derived from dLNs of neonatal mice immunized with HA/CAF01. IL4 expression was significantly higher in mice receiving HA/GLASE. Further, higher expression of IL10 protein levels, albeit not statistically significant, was observed (**Figure 5** **)**, whereas IL21 protein levels remained under detection limit (data not shown). In neonates immunized with HA/CAF01 we also observed higher levels of IFNγ and IL17, that together with the lower levels of IL4, revealed the ability of CAF01 to induce mixed Th1/Th17 responses in neonates (**Figure 5**), as previously observed in adults (24).

These results confirmed that CAF01 and GLA-SE induce different cytokine environments in the dLNs of neonatal mice that are determinant for the quality of the resulting T cell responses.

### Only CAF01 induces adult-like IL21-producing Tfh cells in neonatal mice

The induction of GC and antibody responses is dependent on Tfh cells. Potent Tfh responses rely on high expression of specific functional markers such as BCL6, the master transcriptional regulator of Tfh differentiation, ICOS, and IL21, which is critical for GC formation (40–43).

**Figure 6:**
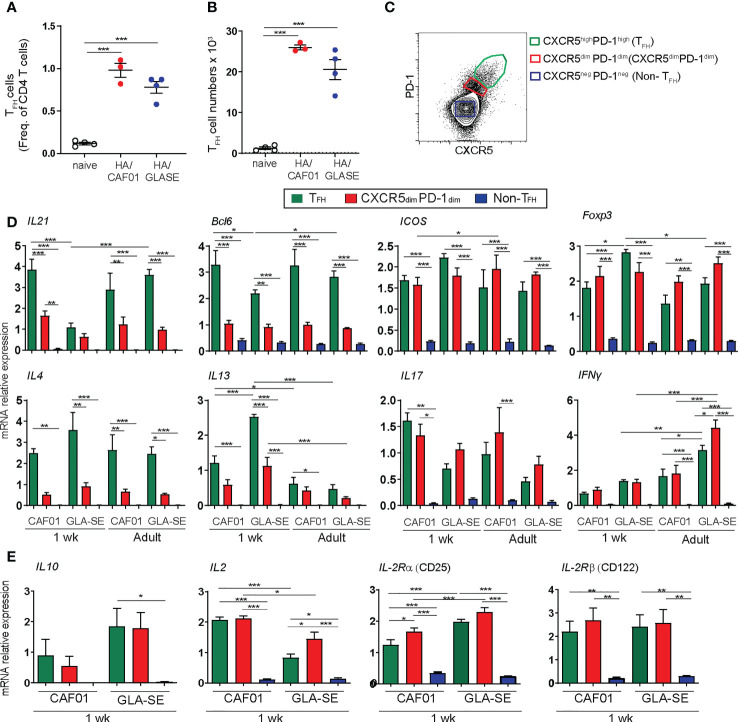
Only CAF01 induces neonatal Tfh cells expressing adult-like levels of IL21. **(A, B)** One week-old CB6F1 mice were immunized s.c. with HA/CAF01 or HA/GLA-SE and dLNs were harvested at day 10 post immunization to quantify Tfh cells by flow cytometry. Graphs report **(A)** frequencies and **(B)** numbers of Tfh cells from a representative experiment. Statistical analyses were performed using the Mann-Whitney U test: ***P < 0.001. **(C-E)** One week-old and adult CB6F1 mice were immunized s.c. with HA/CAF01 or HA/GLA-SE. At day 10 post immunization, the draining LNs were collected to simultaneously isolate highly pure CD4^+^CXCR5^high^PD-1^high^ Tfh cells, CD4^+^CXCR5^dim^PD-1^dim^ effector T cells (CXCR5^dim^PD-1^dim^) and CD4^+^CXCR5^neg^PD-1^neg^ resting T cells (non- Tfh) by flow cytometry cell sorting according to the gates illustrated in panel **(C, D)** mRNA expression levels of *IL21*, *Bcl6*, *ICOS*, *Foxp3*, *IL4*, *IL13*, *IL17* and *IFN-γ* are shown in the three distinct populations. **(E)** mRNA expression levels of *IL10*, *IL2, CD25*, and *CD122* are shown in the three distinct populations in neonatal mice. **(C-E)** The cells obtained from the two dLNs of either 8 neonates/group or 6 adults/group per experiment were pooled before sorting to recover sufficient number of cells for experimentation. Results from 3 independent experiments including data from a total of 24 neonates and 18 adults are shown. Results are expressed as mean + SEM. Two -way ANOVA with multiple comparisons: *P < 0.05, **P < 0.01, ***P < 0.001.

We showed that HA/CAF01 and HA/GLA-SE induced similar frequencies and numbers of Tfh cells in neonatal mice (**Figures 6A, B** **)** and the elicited Tfh cells expressed high levels of BCL6 and ICOS when compared to resting CD4 T cells (**Supplementary Figure 4**) (13). However, there is difference in the capacity of the elicited Tfh cells to produce IL21. To assess this directly, we measured IL21 levels in Tfh cells isolated from the dLNs of neonatal and adult mice immunized with HA/CAF01 or HA/GLA-SE.

CD4^+^CXCR5^high^PD-1^high^ Tfh cells, CD4^+^CXCR5^dim^PD-1^dim^ effector T cells (CXCR5^dim^PD-1^dim^), and CD4^+^CXCR5^neg^PD-1^neg^ resting T cells (non- Tfh) were isolated by fluorescence-activated cell sorting from the draining LNs of neonatal and adult mice 10 days post immunization, according to the gates depicted in **Figure 6C**. The mRNA expression levels of key Tfh - and T helper-associated genes were measured by RT-PCR in the three distinct sorted populations. Comparison between dLNs of neonates and adults indicated that HA/CAF01 induced neonatal Tfh cells that express high, and adult-like level of *IL21* (**Figure 6D**). In contrast, in the Tfh cells of neonates immunized with HA/GLA-SE, *IL21* expression was as low as in CD4^+^CXCR5^dim^PD-1^dim^ effector T cells, with no statistically significant difference compared to the non- Tfh cells that lack *IL21* expression (**Figure 6D**).

The neonatal Tfh cells induced by HA/GLA-SE expressed both *BCL6* and *ICOS* as observed by flow cytometry, and significantly higher levels of *FOXP3* than those induced by HA/CAF01, or in adults (**Figure 6D**). Higher levels of *FOXP3*, typical of T follicular regulatory cells (Tfr) indicate that the Tfh cells induced by GLA-SE in neonates may have a more regulatory phenotype as compared to those elicited by CAF01. Additionally, neonatal Tfh cells elicited by HA/GLA-SE preserved the neonatal Th2-bias, with higher levels of *IL13*, and a trend, albeit not statistically significant, towards higher level of *IL4* and *IL10*, than those induced by HA/CAF01 (**Figure 6D**).

Neonatal Tfh cells are known to secrete large amounts of IL2 (44). The neonatal Tfh and CXCR5^dim^PD1^dim^ effector T cell induced by HA/CAF01 expressed higher levels of IL2 than the ones elicited by HA/GLA-SE, confirming their higher activation status.

In adults, it has been reported that large amounts of autocrine IL2 do not impact Tfh differentiation, due to the IL2 responsiveness regulated by IL-6-STAT3. IL6 is required to inhibit up-regulation of IL-2Rβ (CD122) on adult Tfh cells and maintain their IL2 hypo-responsiveness. Neonatal Tfh cells induced by HA/CAF01 showed lower levels of IL2Rα (CD25) but not of IL2Rβ when compared to the ones elicited by HA/GLASE, although only in the CAF01 group we observed a decreasing trend in IL2Rβ expression between effector T and Tfh cells (**Figure 6E**). Further studies are needed to explore the possible role of IL6 in maintaining IL-2 hyporesponsiveness in neonatal Tfh cells.

In the CAF01 group, neonatal Tfh cells also expressed higher *IL13* levels than their adult counterparts, and high level of *IL17*. In neonatal Tfh cells we observed similar levels of expression of *IFNγ* between CAF01 and GLA-SE, while in adult mice the cells elicited by GLA-SE expressed higher levels of *IFNγ*, as shown previously (24).

At the RNA level, the T helper cells elicited by HA/GLA-SE in neonates expressed similar levels of *IFN*γ to those elicited by HA/CAF01, but higher level of *IL13* and *IL4*, confirming a more pronounced Th2 profile.

Altogether, these findings revealed the ability of a single dose of a CAF01-containing vaccine to induce Tfh cells expressing high levels of IL21, which is a key step for the induction of GC B cell responses.

### Fully functional Tfh cells are a key requirement for the development of neonatal GC reactions following immunization with CAF01

We sought to confirm the functionality of CAF01-induced neonatal Tfh cells by probing their ability to promote fully mature B cell differentiation into GC B cells.

To this aim, we used the well-described model of B and T cell responses against the protein antigen hen egg lysozyme (HEL)-conjugated ovalbumin (OVA), which triggers robust T-dependent responses by transgenic HEL-specific SW_HEL_ B cells (25, 32).

**Figure 7:**
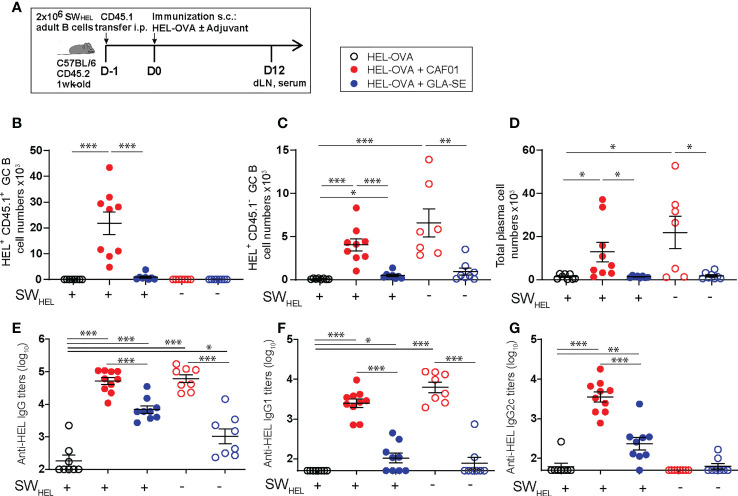
The initiation of neonatal GC reactions requires fully functional Tfh cells. **(A)** Experimental schedule of the adult SWHEL B cells adoptive transfer. Naïve congenic 1-week old C57BL/6 recipient mice received each 2 x 10^6^ adult CD45.1^+^ B cells by intraperitoneal injection. Recipient and control neonatal mice were immunized s.c. the following day with 20 µg HEL-OVA formulated in CAF01, GLA-SE or PBS as control. Serum samples and inguinal draining LNs were collected 12 days post immunization. **(B–D)** The formation of HEL-specific GCs and plasma cells was measured by flow cytometry in the draining LNs of immunized neonates; graphs show the number of **(B)** SWHEL HEL^+^ CD45.1^+^ GL7^+^ CD95^+^ GC B cells, **(C)** endogenous HEL^+^ CD45.1^-^ GL7^+^ CD95^+^ GC B cells, and **(D)** total frequencies of B220int/lowCXCR4^+^CD138^+^ PCs. **(E-G)** HEL-specific serum **(E)** IgG, **(F)** IgG1, and **(G)** IgG2c titers were measured by ELISA. Dots show values per individual mouse (N≥ 6 per group) whereas black bars indicate means ± SEM. Statistical analysis were performed using the Mann-Whitney U test: *P < 0.05, **P < 0.01, ***P < 0.001, ****P < 0.0001. Data were pooled from at least two independent experiments.

We adoptively transferred adult HEL-specific CD45.1^+^ SW_HEL_ B cells into congenic CD45.2^+^ C57BL/6 neonatal mice. Recipient and control mice were immunized s.c. 1 day later with HEL-OVA alone, or formulated in either GLA-SE or CAF01 (**Figure 7A** **)**. Twelve days post immunization, a time at which CAF01 induces high neonatal endogenous GC B cell numbers (13), we measured both adult HEL-specific CD45.1^+^ GC B cells and endogenous neonatal HEL-specific CD45.1^-^ GC B cell responses by flow cytometry (gating strategy in **Supplementary Figure 5**). Adult CD45.1^+^ SWHEL B cells strongly expanded and formed HEL^+^CD45.1^+^ GCs in the draining LNs of CAF01—but not of GLA-SE—immunized neonates (**Figure 7B**). After SW_HEL_ B cell transfer, CD45.1^+^ GC B cells increased only slightly in neonates immunized with HEL-OVA/GLA-SE compared to mice that received unadjuvanted HEL-OVA (**Figure 7B**). Consistently with our previous data with HA antigen (13), only CAF01 elicited high endogenous HEL^+^ CD45.1^-^ GC B cell responses in neonates (**Figure 7C**). In line with the higher GC B cell responses, the development of B220^dim^CD138^+^ plasma cells was only observed in neonates immunized with HEL-OVA/CAF01 (**Figure 7D**).

The transfer of adult SWHEL B cells did not affect the level of HEL-specific IgG antibody titers generated by HEL-OVA/CAF01, suggesting that the environment promoted by CAF01 is sufficient to reach an optimal antibody production capacity by the endogenous and immature neonatal B cells. In contrast, HEL-OVA/GLA-SE slightly increased HEL-specific IgG responses after transfer, which correlated with the presence of few HEL^+^CD45.1^+^ GC B cells (**Figure 7E**).

Interestingly, high level of IgG2c was only observed in mice adoptively transferred with adult B cells upon immunization with HEL-OVA/CAF01, suggesting that CAF01 can generate a Th1/Th17-prone environment but switching to cytotoxic isotypes may require B cells to be more mature (adult) (**Figures 7F, G** **)**. Interestingly, in this model CAF01 induced more Tfh cells than GLA-SE at day 12. Tfh cells induced by CAF01 also expressed higher levels of Bcl6, but not ICOS, in agreement with data presented in **Figure 6D**. GC B cells promote Tfh expansion and may be responsible for higher and sustained Tfh responses in neonatal mice receiving CAF01. A significantly higher proportion of Foxp3+GITR+ Tfr cells was observed in neonates following HEL-OVA adjuvantation with GLA-SE than CAF01 (**Supplementary Figure 6**) in line with our previous observation at the transcript level.

Altogether, our data show the ability of CAF01 at inducing fully functional Tfh cells and thereby, *bona fide* GC responses in neonates.

## Discussion

Our genome-wide transcriptional analysis showed that both CAF01 and GLA-SE induced significant changes in the expression of genes encoding cytokines and chemokines, interferon responses, JAK-STAT and TLR signaling pathways, and CD molecules in the dLNs of neonatal mice at one- and 7-days post-immunization. Key differences were observed at day 7 post-injection, with the two adjuvants inducing a different environment in dLNs, which is critical for the generation of functional Tfh and the subsequent B cell responses.

In-depth comparison between the CLR-based CAF01 adjuvant and the TLR-based GLA-SE adjuvant revealed the ability of CAF01 to induce high levels of IL6 and IL12B in dLNs of neonatal mice 7-10 days post immunization. This selective production of cytokines correlated with the induction of functional IL21-producing Tfh cells, able to activate neonatal and adult B cells to induce primary GC B cells and IgG antibody responses.

Cytokine signaling is crucial for Tfh generation and differentiation, with IL6, IL12 and IL-21 playing a critical role in adults. APC-derived IL6 and IL12 stimulate IL21 production by CD4+ T cells and promote Tfh differentiation. IL12 promotes expression of CXCR5, ICOS, PD-1, BCL6 and IL21 in activated CD4^+^ T cells (45, 46). IL6 significantly increases BCL6 and IL21 production and antibody production by B cells (47). At day 7–10 post-injection, increased levels of those cytokines were only observed in dLNs of neonatal mice receiving HA/CAF01, suggesting that these cytokines contribute to the induction of neonatal GC-Tfh cells.

High IL6 levels in dLNs, together with IL12 and IL21 levels, correlated with the induction of functional Tfh cells expressing adult-like levels of IL21 and with potent primary B cell responses. Our data contradicts the general negative role of endogenous IL6 on neonatal vaccine responses reported by Yang J. et al. (48). Higher levels of IL6 were found in dLNs of neonatal mice 24h after HA/GLA-SE immunization, but not at day 7 - pointing to the importance of the kinetic of IL6 production in controlling Tfh/B cell induction. Neither i.p. nor s.c. IL6 administration starting from day 4 post HA/GLA-SE immunization rescued primary neonatal GC B cell responses (not shown), suggesting that IL-6 alone is not sufficient. Rather, a combination of factors seems required to create the appropriate environment to generate efficient GC-Tfh reactions.

In addition to Tfh cells, Tfr cells reside within secondary lymphoid tissues, where they inhibit GC B cell responses (49, 50). Tfr cells express surface receptors shared by Tfh cells, such as CXCR5, ICOS and PD-1, and even BCL6 (51), but are typically defined by the co-expression of FOXP3 and CD25. In neonates, functional Tfh cells, expressing high levels of IL-21 and low levels of Foxp3 similar to adults, were only elicited by CAF01.

Neonatal Tfh cells induced by HA/GLA-SE lacked expression of IL21 and expressed lower levels of Bcl6 and higher levels of Foxp3 and CD25, the latter being hallmark features of the Tfr rather than the Tfh phenotype.

Our study suggests that the induction of functional Tfh cells with the help of adequate adjuvants is critical to elicit potent neonatal B cell responses following vaccination. An environment rich in IL-6, IL-12 and IL-21 with low levels of IL-10 and IL-4 favors Tfh over Tfr phenotype.

It remains to be established how CLR activation could lead to the generation of such environment in dLNs. Binding of TDB, included in CAF01, to macrophage−inducible C−type lectin (MINCLE) triggers the FcRγ-Syk-Card9 pathway for APC activation (52). This signaling pathway activates TLR−independent production of cytokines such as tumor necrosis factor (TNF) and IL6. Stimulation with TDB was shown to increase IL-6 secretion and MINCLE expression in healthy human PBMCs (53). IL-6 produced by follicular dendritic cells promoted germinal center reactions, IgG antibody responses and somatic hypermutation in adults (36, 54). Follicular DCs are therefore a plausible local source of IL-6 in neonatal dLNs, although their role is technically difficult to assess due to their very low numbers in neonatal dLNs. Further, in adult lymph nodes, a subset of CD11c^high^ DCs co-localizing with plasma cells in extrafollicular foci are high producers of IL-6 (55). Whether this cell subset is present in neonates and could be a target of CAF01 remains to be investigated.

Interestingly, in neonatal mice the presence of adult B cells was required to generate IgG2c antibody responses (Th1-related IgG isotype) revealing that additional limiting factors may be at play in neonates, and that these factors need to be circumvented by vaccine adjuvant combinations for induction of optimal B cell responses in this age group.

Altogether our data confirm the ability of CLR agonist-containing adjuvants to induce potent primary B cell responses in a neonatal environment, and demonstrate that this property is mediated through the induction of IL-21 producing adult-like Tfh cells activating B cells to form GC reactions. These results call for further investigation to test the potency and safety of CLR-agonist adjuvants in large young animals and children. The data presented in this study inform the rational design of next generation vaccines able to mount protective immunity against infections in early life.

## Data availability statement

The original contributions presented in the study are publicly available. The microarray data have been deposited to the Gene Expression Omnibus under accession number GSE226513.

## Ethics statement

The animal study was reviewed and approved by the Geneva veterinary office (authorization GE/05/16) and conducted under relevant Swiss and European guidelines.

## Author contributions

MV, P-HL, AH and C-AS designed the research and interpreted the data; MV, BM-G, EM, JP, TO, AD and DP performed the experiments, analyzed data and/or provided intellectual input. MÖ and SL performed microarray data analysis. DC, PA, and OH provided reagents, and intellectual input. MV and C-AS wrote the manuscript. All authors reviewed the manuscript. All authors contributed to the article and approved the submitted version.
