# Supplementary material for: C-type lectin receptor agonists elicit functional IL21-expressing Tfh cells and induce primary B cell responses in neonates
Source: Front Immunol. 2023 Mar 31;14:1155200. doi: 10.3389/fimmu.2023.1155200 (PMC10102809; doi:10.3389/fimmu.2023.1155200)
Supplement: Supplementary file 1 [file DataSheet_1.pdf]

Supplementary Material

Original Article

C-type lectin receptor agonists elicit functional IL21-expressing Tfh cells and induce primary B cell responses in neonates.

Maria Vono[1*], Beatris Mastelic-Gavillet[1], Elodie Mohr[1], Malin Östensson[2], Josefine Persson[2], Thorunn A. Olafsdottir[3], Sylvain Lemeille[4], David Pejoski[1], Oliver Hartley[4], Dennis Christensen[5], Peter Andersen[5], Arnaud M. Didierlaurent[1], Ali M. Harandi[2,6], Paul-Henri Lambert[1] and Claire-Anne Siegrist[1]

*** Correspondence:** Maria Vono: maria.vono@unige.ch

1 Supplementary Figures and Tables

1.1 Supplementary Figures

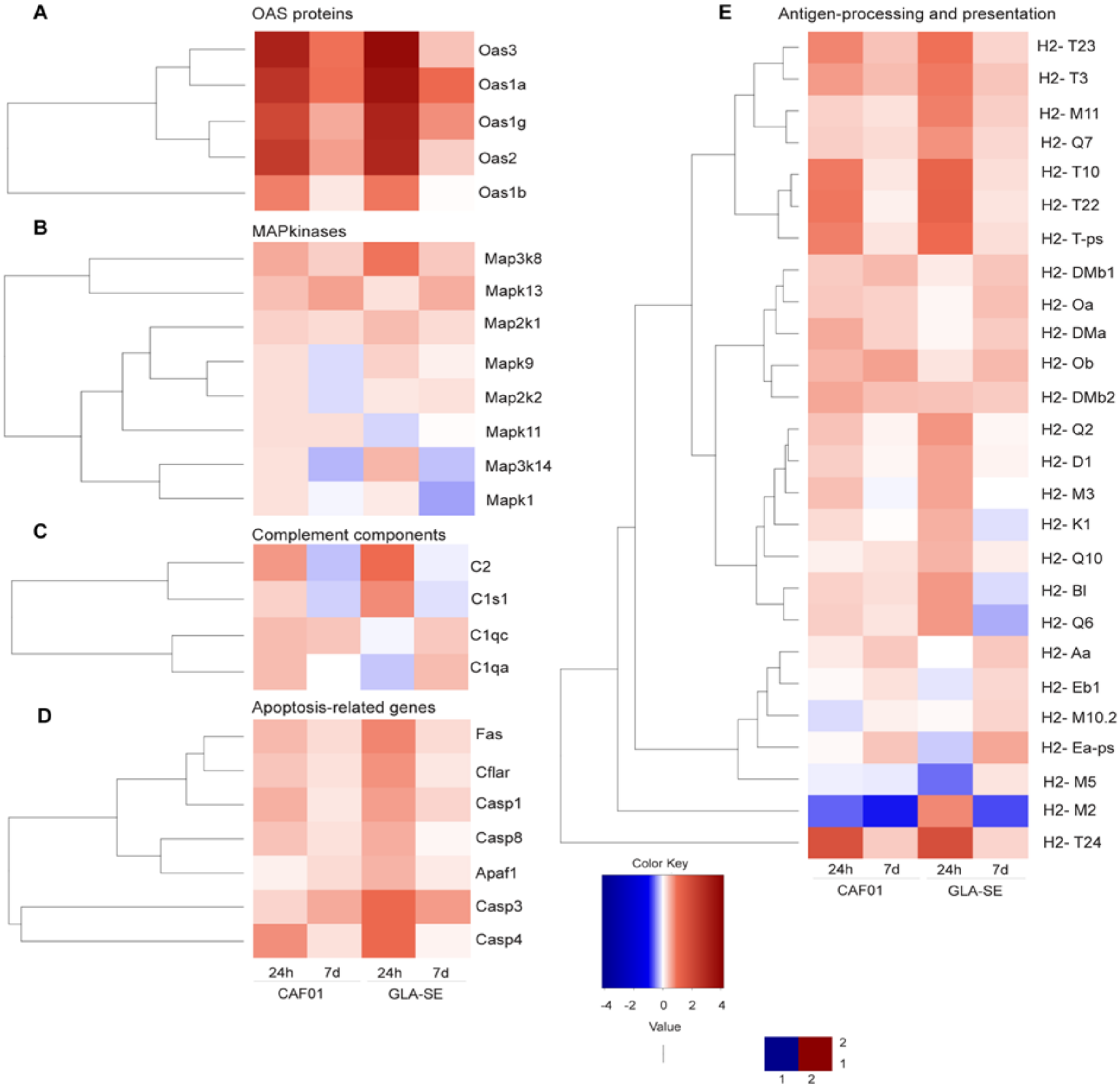

Supplemental Figure 1. Transcriptomic profiling of whole draining LNs from neonatal mice immunized with HA/CAF01 or HA/GLA-SE. Related to Figure 1. (A-E) Groups of CB6F1 neonatal mice were immunized s.c. with HA alone or formulated with either CAF01 or GLA-SE. The two draining LNs were collected 24 h and 7 days post-immunization to perform microarray analysis. Transcriptomic profiles resulting from the leading-edge analysis are shown. Heat maps show analysis of the expression profiles of differentially expressed genes obtained by comparisons of HA/CAF01 vs HA and HA/GLASE vs HA and organized in the following categories: (**A**) OAS proteins, (**B**) MAPKinases, (**C**) complement components, (**D**) apoptosis-related genes, and (**E**) antigen processing and presentation.

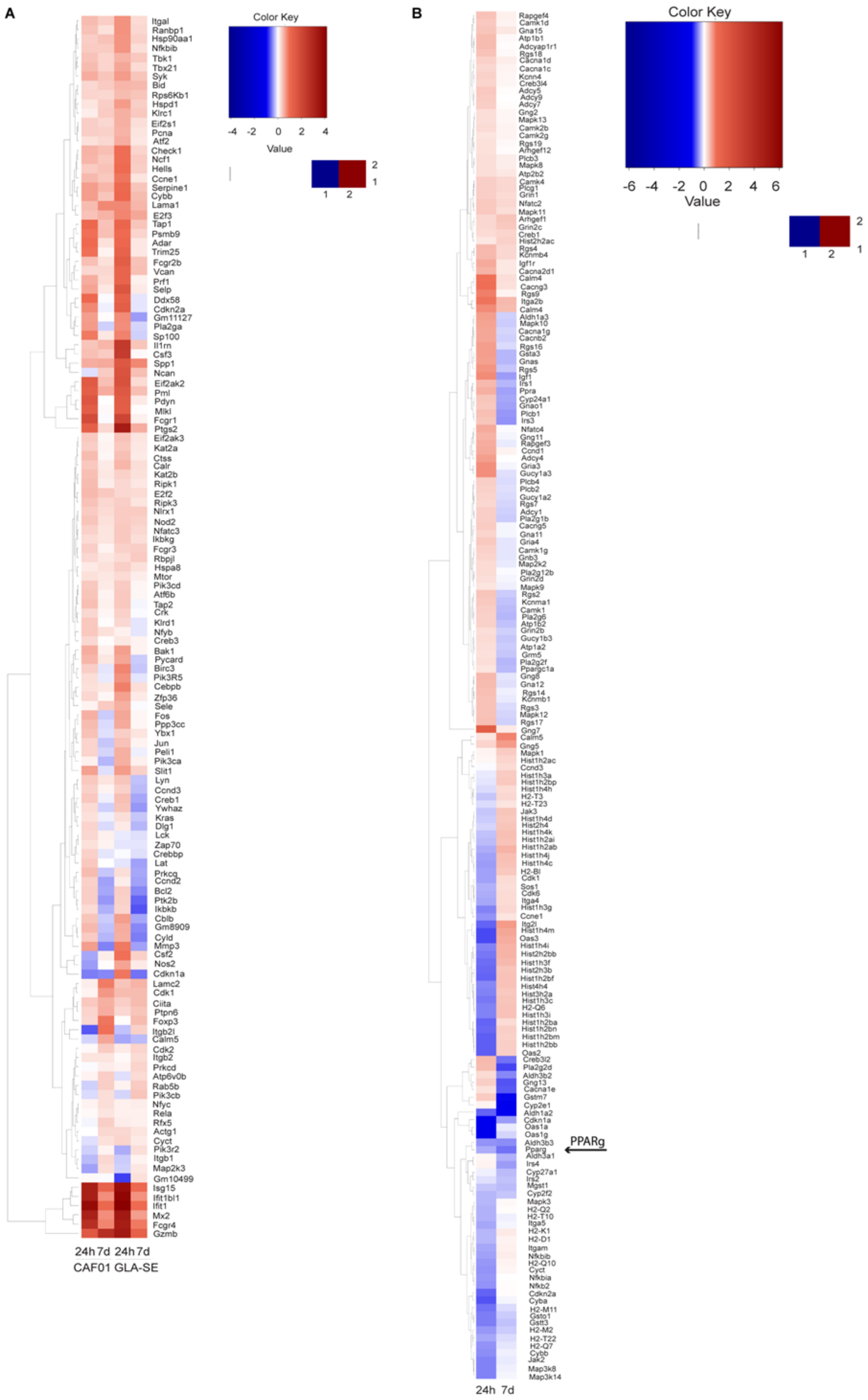
A
Color Key
-4 -2 0 2 4
Value
1 2
2
1
Itgal
Ranbp1
Hsp90aa1
Nfkbib
Tbk1
Tbx21
Syk
Bid
Rps6Kb1
Hspd1
Klrc1
Eif2s1
Pcna
Atf2
Check1
Ncf1
Hells
Ccne1
Serpine1
Cybb
Lama1
E2f3
Tap1
Psmb9
Adar
Trim25
Fcgr2b
Vcan
Prf1
Selp
Ddx58
Cdkn2a
Gm11127
Pla2ga
Sp100
Il1rn
Csf3
Spp1
Ncan
Eif2ak2
Pml
Pdyn
Mlkl
Fcgr1
Ptgs2
Eif2ak3
Kat2a
Ctss
Calr
Kat2b
Ripk1
E2f2
Ripk3
Nlrx1
Nod2
Nfatc3
Ikbkg
Fcgr3
Rbpjl
Hspa8
Mtor
Pik3cd
Atf6b
Tap2
Crk
Klrd1
Nfyb
Creb3
Bak1
Pycard
Birc3
Pik3R5
Cebpb
Zfp36
Sele
Fos
Ppp3cc
Ybx1
Jun
Peli1
Pik3ca
Slit1
Lyn
Ccnd3
Creb1
Ywhaz
Kras
Dlg1
Lck
Zap70
Crebbp
Lat
Prkcq
Ccnd2
Bcl2
Ptk2b
Ikbkb
Cblb
Gm8909
Cyld
Mmp3
Csf2
Nos2
Cdkn1a
Lamc2
Cdk1
Ciita
Ptpn6
Foxp3
Itgb2l
Calm5
Cdk2
Itgb2
Prkcd
Atp6v0b
Rab5b
Pik3cb
Nfyc
Rela
Rfx5
Actg1
Cyct
Pik3r2
Itgb1
Map2k3
Gm10499
Isg15
Ifit1bl1
Ifit1
Mx2
Fcgr4
Gzmb
24h 7d 24h 7d
CAF01 GLA-SE
B
Color Key
-6 -4 -2 0 2 4 6
Value
1 2
2
1
PPARg
24h 7d

Supplemental Figure 2. Transcriptomic profiling of whole draining LNs from neonatal mice immunized with HA/CAF01 or HA/GLA-SE. Related to Figure 1 and Figure 2.
(A, B) Groups of neonatal CB6F1 mice were immunized s.c. with HA alone, or formulated with either CAF01 or GLA-SE. Transcriptomic profiles resulting from the leading edge analysis are shown. (**A)** Heat map shows analysis of the expression profiles of all remaining DEGs identified by the comparisons between HA/CAF01 vs HA and HA/GLASE vs HA, and that did not fit into Figures 1 and S1. (**B**) Heat map shows analysis of the expression profiles of all remaining DEGs identified by comparing HA/CAF01 vs HA/GLASE.

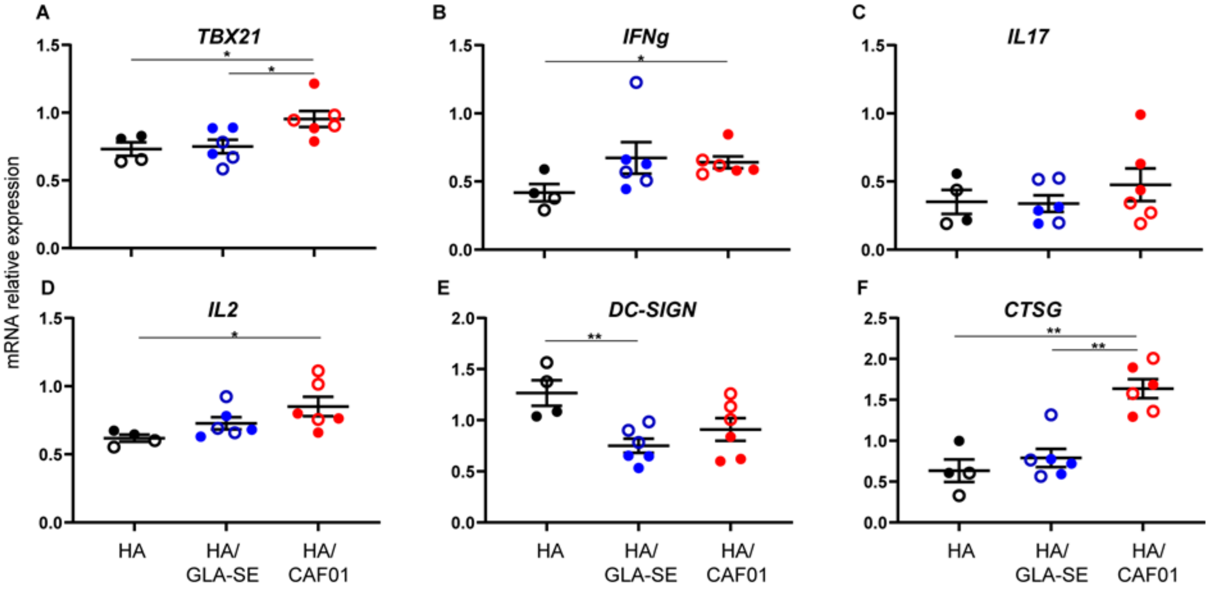

Supplemental Figure 3. Expression levels of selected genes detected by RT-PCR. Related to Figure 4.

(A-F) One week-old CB6F1 mice were immunized s.c. with HA/PBS, HA/CAF01 or HA/GLA-SE and the 2 draining LNs were collected at day 7 or 10 post-immunization. RNA from total dLNs was used to measure by RT-PCR the expression level of selected DEGs identified by microarray-based gene expression profiling. mRNA expression level of (**A**) *TBX21*, (**B**) *IFNg*, (**C**) *IL17*, (**D**) *IL2*, (**E**) *DC-SIGN*, and (**F**) *CTSG* are shown. Dots show values per individual mouse (N ≥ 4 per group) whereas black bars indicate means ± SEM. Statistical analysis were performed using the Mann-Whitney U test: *$P < 0.05$, **$P < 0.01$. Data from day 7 (open circle) and 10 (filled circle) post-immunization were pooled. Data from one representative experiment out of two.

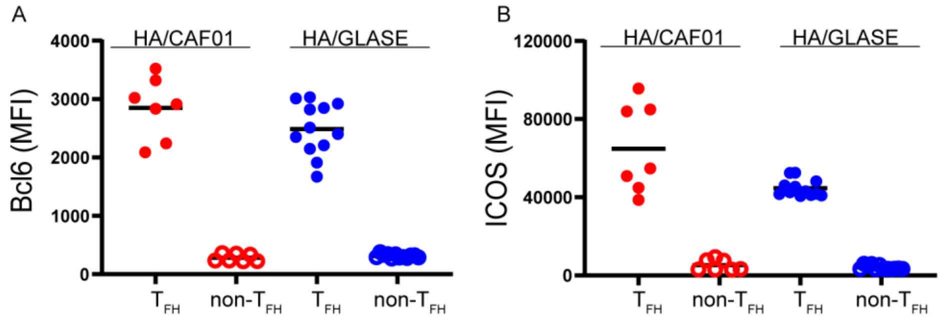

Supplemental Figure 4. T follicular helper cells highly express Bcl6 and ICOS. Related to Figure 6.

(A, B) One-week-old CB6F1 mice received a single dose of HA/CAF01 or HA/GLASE. Draining LNs were harvested to quantify T follicular helper (T_{FH}) cells and measure the level of expression of Bcl6 and ICOS by flow cytometry at day 10 post-immunization. Geometric mean fluorescence intensity (MFI) values of (**A**) Bcl6 and (**B**) ICOS on T_{FH} and the corresponding non-T_{FH} cells. Dots show values per individual mouse whereas lines indicate means. N ≥7 mice/group. Data are representative of at least 3 independent experiments.

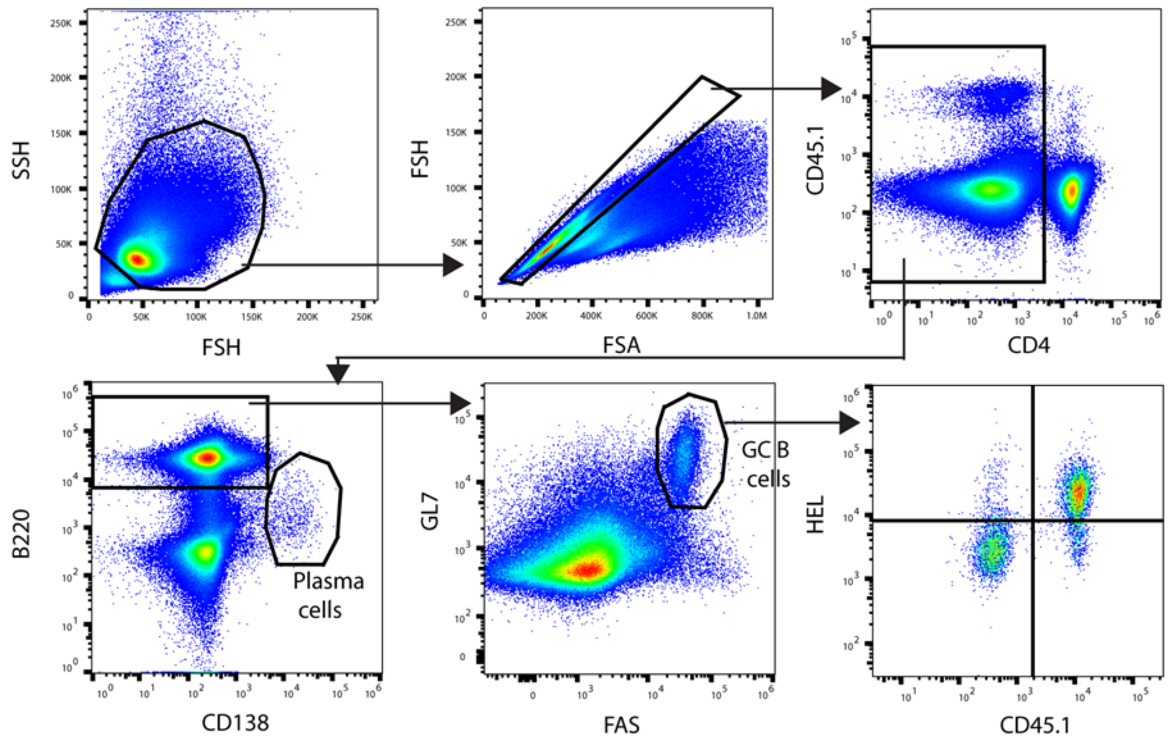

Supplemental Figure 5. Gating strategy of draining lymph node-derived cells from the transfer experiment. Related to Figure 7.
Lymph node single cell suspensions were prepared and analyzed by flow cytometry, applying the depicted gating strategy.

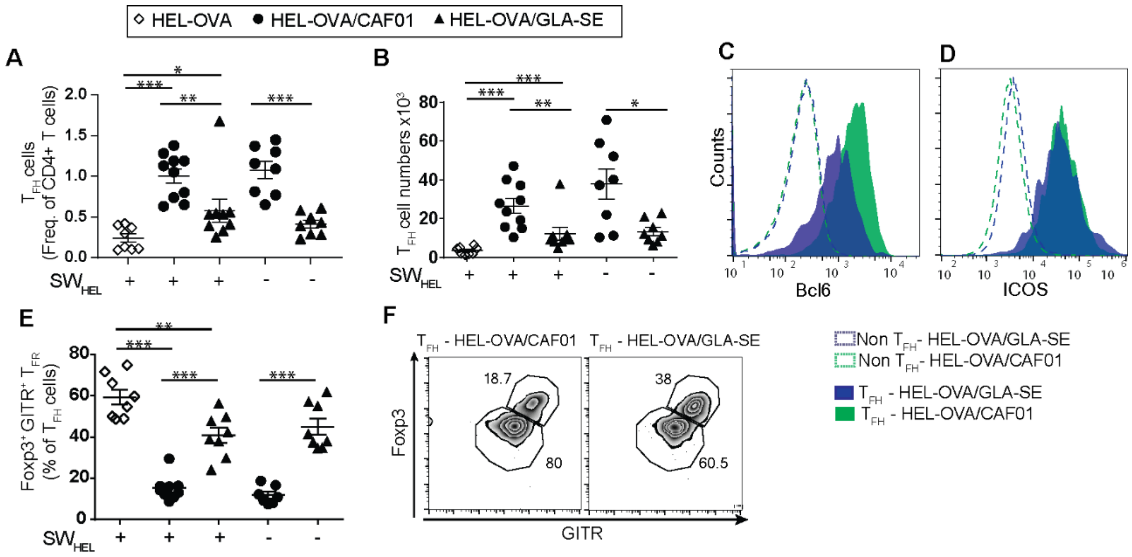

Supplemental Figure 6. Characterization of the neonatal Tfh responses elicited by HEL-OVA/CAF01 and HEL-OVA/GLA-SE. Related to Figure 7.

(**A-F**) Naïve congenic 1-week old C57BL/6 recipient mice received each 2 x 10^6 adult CD45.1$^+$ B cells by intraperitoneal injection. Recipient and control neonatal mice were immunized s.c. the following day with HEL-OVA alone or formulated in either CAF01 or GLA-SE. Inguinal draining LNs were collected 12 days post immunization. **(A, B)** Graphs report **(A)** frequencies and **(B)** numbers of CXCR5^{high}PD-1^{high} Tfh cells. **(C, D)** Representative histograms show the expression levels of **(C)** Bcl6 and **(D)** ICOS in the indicated cell populations. **(E)** Graph shows Foxp3$^+$GITR$^+$ Tfr cells expressed as percentages of CXCR5^{high}PD-1^{high} Tfh cells. **(F)** Representative dot plots show percentages of Tfr in dLN cells from neonatal mice immunized with either HEL-OVA/CAF01 or HEL-OVA/GLASE. Dots show values per individual mouse (N≥ 6 per group) whereas black bars indicate means ± SEM. Statistical analysis were performed using the Mann-Whitney U test: *P < 0.05, **P < 0.01, ***P < 0.001.

1.2 Supplementary Tables

Supplementary Table 1. Primers used in this study.

Gene	Forward (5´- 3´)	Reverse (3´- 5´)
IL6	ACACATGTTCTCTGGGAAATCGT	AAGTGCATCATCGTTGTTCATACA
IL12B	AGACCCTGCCCATTGAACTG	GAAGCTGGTGCTGTAGTTCTCATATT
IL4	GAGCTGCAGAGACTCTTTCG	ACTCATTCATGGTGCAGCTTA
IL10	TTTGAATTCCCTGGGTGAGAA	GCTCCACTGCCTTGCTCTTATT
IL13	ACAGGACCCAGAGGATATTGCA	GGGAGGCTGGAGACCGTAGT
IL1B	ACCCTGCAGCTGGAGAGT	CCATCTTCTTCTTTGGGTATTGCTT
IL21	TCAGCTCCACAAGATGTAAAGGG	GGGCCACGAGGTCAATGAT
GZMB	GACCTTGTCTCTGGCCTCCA	ATGTCCCCCGATGATCTCC
CTSG	TGTTGACCTTTATTCTACTCCAAGGA	CTCCAATGATCTTCCCTGCC
Bcl6	TGAAATCTGTGGCACTCG	CAA ATG AAG TCG CAG TTG G
ICOS	GGAACCTTAGTGGAGGATATTTGC	TGGCAGCAGAGCTGGGA
Foxp3	TTATCCGATGGGCCATCCT	GCGAGTAAACCAATGGTAGATTTCA
IL17a	TGGACTCTCCACCGCAATG	GCACTGAGCTTCCCAGATCAC
IFNγ	CAAGCGGCTGACTGAACTCA	CACTGCAGCTCTGAATGTTTCTTATT
IL2	CAGCAATATCAGAGTAACTGTTGTAAA ACT AA	CGAATTGGCACTCAAATGTGTT
IL2Ra	GCAGCAACTGCCAGTGCA	AACTTGCTTTCTCGATTTGTCATG
IL2Rb	CTGAGCTCCCAGCATGGG	GACAGGCGAGGAGAGCCA

2 Data Availability Statement

The microarray data have been deposited to the Gene Expression Omnibus under accession number GSE226513. Requests to access the other datasets should be directed to MV, maria.vono@unige.ch.
